# Complete Genome Sequence of *Pseudomonas aeruginosa* Strain 8380, Isolated from the Human Gut

**DOI:** 10.1128/genomeA.00520-15

**Published:** 2015-05-21

**Authors:** Yu-ki Ichise, Takehide Kosuge, Maki Uwate, Taiji Nakae, Hideaki Maseda

**Affiliations:** a Institute of Technology and Science, Tokushima University, Tokushima, Japan; b DDBJ Center, National Institute of Genetics, Mishima, Japan; c Graduate School of Medical Science, Kitasato University, Sagamihara, Japan

## Abstract

*Pseudomonas aeruginosa* shows multidrug resistance, which is mainly attributable to its expression of xenobiotic efflux pumps. However, it is unclear how silent pumps are expressed in clinical isolates. Here, we sequenced the complete genome of *P. aeruginosa* strain 8380, which was isolated from a human gut.

## GENOME ANNOUNCEMENT

*Pseudomonas aeruginosa*, a prevalent Gram-negative pathogen, is a key causative agent of acute and chronic infections in immunocompromised hosts (1). This organism shows multidrug resistance, which is mainly attributable to the interplay of low outer membrane permeability and the intrinsic expression of the resistance-nodulation-cell division (RND)-type efflux pumps (2, 3). A multidrug-resistant mutant, the NfxC-type mutant, shows resistance to chloramphenicol and fluoroquinolones via the induction of one of the most clinically significant RND-type efflux pumps, MexEF-OprN, and to imipenem via the reduction of OprD porin on cells (4, 5). This phenotype is caused by MexT, which is one of major global regulators in *P. aeruginosa* (5–7). We previously reported that the activation of MexT in clinically isolated 8380 differs from that of PAO1 (7, 8). MexT is inactive in PAO1 and 8380 cells under laboratory conditions because PAO1 has an impaired *mexT* locus, whereas 8380 has an unimpaired *mexT* locus and a higher activity of the MexT repressor, *mexS*_8380_ (7, 8). Thus, a mutation in *mexS* is required for activation of MexT and expression of MexEF-OprN in 8380 (8). We have also reported that the sequence of *mexS*_8380_ contains an important substitution, G745A, which changes an amino acid, and D249N, which increases its activity compared with that of *mexS*_PAO1_ (8).

Here, we announce the complete genome sequence of the clinical isolate *P. aeruginosa* strain 8380. The 8380 genome was sequenced using a Pacific Biosciences PacBio RSII sequencer. A total of 75,207 reads, averaging 8,691 bp in length, were obtained for a total of 653,628,053 bases of sequence. Genome assembly was performed with the RS_HGAP_Assembly.3 protocol, and a single contig was obtained. The assembled sequence of the 8380 genome comprised a single circular chromosome of 6,613,159 bp. The average GC content of the chromosome was 66.2%, which is consistent with other *P. aeruginosa* strains previously sequenced. Automated genome annotation was carried out by means of both Prokka and RAST (9). In addition to these automated annotations, protein sequences were queried against the Swiss-Prot database using BLASTp, and the annotation was manually curated. The complete 8380 genome has 6,210 protein-coding sequences, 63 tRNA genes, 12 rRNA genes, and a single transfer-messenger RNA (tmRNA) gene. Twelve RND-type efflux pumps, which were discovered in PAO1, were predicted in the 8380 genome (10, 11). Among them, MexAB-OprM, MexCD-OprJ, and MexXY efflux pumps are overexpressed by one or more mutations in the repressors *mexR*, *nfxB*, and *mexZ*, respectively, each of which contributes to antibiotic resistance (12–14). *mexR* and *nfxB* in 8380 each had one synonymous substitution, C67A or T555G, respectively, and *mexZ* contained no mutations compared with those in PAO1. Moreover, 8380 cells showed antibiotic susceptibility comparable with that of PAO1 cells (4, 8). More detailed analyses of the 8380 genome are ongoing.

### Nucleotide sequence accession number.

The complete 8380 genome sequence had been deposited in DDBJ under the accession no. AP014839.
